# Elucidation of the outer membrane proteome of *Salmonella enterica *serovar Typhimurium utilising a lipid-based protein immobilization technique

**DOI:** 10.1186/1471-2180-10-44

**Published:** 2010-02-11

**Authors:** Darren Chooneea, Roger Karlsson, Vesela Encheva, Cath Arnold, Hazel Appleton, Haroun Shah

**Affiliations:** 1 Department for Bioanalysis and Horizon Technologies, Health Protection Agency Centre for Infections, 61 Colindale Avenue, London NW9 5EQ, UK; 2 Department of Chemistry, University of Gothenburg, Kemivägen 10 SE-412 96 Göteborg, Sweden; 3 Virus Reference Department, Health Protection Agency Centre for Infections, 61 Colindale Avenue, London NW9 5EQ, UK; 4 Laboratory of the Government Chemist, Queens Rd, Teddington, Middlesex, TW11 0LY, UK

## Abstract

**Background:**

*Salmonella enterica *serovar Typhimurium (*S*. Typhimurium) is a major cause of human gastroenteritis worldwide. The outer membrane proteins expressed by *S*. Typhimurium mediate the process of adhesion and internalisation within the intestinal epithelium of the host thus influencing the progression of disease. Since the outer membrane proteins are surface-exposed, they provide attractive targets for the development of improved antimicrobial agents and vaccines. Various techniques have been developed for their characterisation, but issues such as carryover of cytosolic proteins still remain a problem. In this study we attempted to characterise the surface proteome of *S*. Typhimurium using Lipid-based Protein Immobilisation technology in the form of LPI™ FlowCells. No detergents are required and no sample clean up is needed prior to downstream analysis. The immobilised proteins can be digested with proteases in multiple steps to increase sequence coverage, and the peptides eluted can be characterised directly by liquid chromatography - tandem mass spectrometry (LC-MS/MS) and identified from mass spectral database searches.

**Results:**

In this study, 54 outer membrane proteins, were identified with two or more peptide hits using a multi-step digest approach. Out of these 28 were lipoproteins, nine were involved in transport and three with enzyme activity These included the transporters BtuB which is responsible for the uptake of vitamin B_12_, LamB which is involved in the uptake of maltose and maltodextrins and LolB which is involved in the incorporation of lipoproteins in the outer membrane. Other proteins identified included the enzymes MltC which may play a role in cell elongation and division and NlpD which is involved in catabolic processes in cell wall formation as well as proteins involved in virulence such as Lpp1, Lpp2 and OmpX.

**Conclusion:**

Using a multi-step digest approach the LPI™ technique enables the incorporation of a multi-step protease work flow ensuring enough sequence coverage of membrane proteins subsequently leading to the identification of more membrane proteins with higher confidence. Compared to current sub-cellular fractionation procedures and previous published work, the LPI™ technique currently provides the widest coverage of outer membrane proteins identified as demonstrated here for *Salmonella *Typhimurium.

## Background

The Gram-negative bacterial pathogen *Salmonella enterica *serovar Typhimurium (*S*. Typhimurium) is a leading cause of human gastroenteritis worldwide. It has the ability to infect a broad range of hosts such as poultry, pigs, cattle, rodents and human and the severity of disease is sometimes determined by the type of host infected [1]. For example in mice *S*. Typhimurium exhibits symptoms similar to those of human typhoid, while in humans it causes classical non-typhoidal gastroenteritis [2,3].

The genome of *S*. Typhimurium contains a large number of prominent genes that code for virulence factors which are non-existent in non-pathogenic strains. Regions of the genome that code for these virulence factors are known as pathogenicity islands. *S*. Typhimurium possesses two major islands, which are known as *Salmonella *pathogenicity island 1 (SPI1) and *Salmonella *pathogenicity island 2 (SPI2). These islands encode for two different type III secretion systems (TTSS) [4]. The TTSS is responsible for enabling pathogenic *Salmonella *to transfer virulence factors into the host, allowing it to invade and hijack the host cellular processes [4,5]. SPI1 encodes for the TTSS1, responsible for the invasion of the host's intestinal cells, while SPI2 encodes for the TTSS2, responsible for the survival and proliferation of the bacteria within the host cells [6]. Overall, the TTSS consists of more than 20 proteins including soluble cytoplasmic proteins, integral membrane proteins and outer membrane proteins [5].

The outer membrane proteins are influential in how bacteria interact with each other and with its immediate environment and are actively involved in both the uptake of nutrients and the transport of toxic by-products out of the cell [7]. More importantly, these surface exposed proteins play a critical role in pathogenic processes such as motility, adherence and colonisation of the host cells, injection of toxins and cellular proteases, as well as the formation of channels for the removal of antibiotics (antibiotic resistance) [8,9]. Therefore these functions make outer membrane proteins attractive targets for the development of antimicrobial drugs and vaccines [10,11]. However, it is well documented that the isolation and characterisation of outer membrane proteins has been fraught with difficulty for use in conventional proteomic techniques such as 2D gel electrophoresis (2D GE) due to their association with the membrane or peptidoglycan and relative low abundance when compared to the whole cell complex [7,8,12]. Work carried out by Molloy *et al *attempted to characterise OMPs using 2D GE with the addition of the zwitterionic detergent Amidosulfobetaine-14 (ASB-14) in the rehydration buffer with some degree of success [13]. In addition, several strategies have been developed to try and enrich samples in favour of outer membrane proteins based on differential solubilisation using detergents such as Triton X-100 [14] and sarcosyl [15], chemical enrichment such as sodium carbonate [13] and surface labelling such as biotinylation [16,17]. However, each strategy fails to remove all contaminants such as cytosolic and ribosomal proteins.

New gel-free proteomic approaches such as two dimensional liquid chromatography - tandem mass spectrometry (2D-LC-MS/MS) have been developed for the downstream analysis of complex protein mixtures and are able to overcome the limitations gel based proteomics face especially when dealing with membrane associated proteins [18]. However, these new methods do not focus on preliminary sample preparation where the outer membrane proteins are separated from the rest of the cell protein complex prior to mass spectrometry analysis.

In this study, we have utilised a novel method using lipid-based protein immobilization (LPI) technique to elucidate the outer membrane protein of *S*. Typhimurium. The LPI™ FlowCell is a single use device with a membrane-attracting surface that allows for the immobilisation of intact proteoliposomes (phospholipid vesicle incorporating membrane proteins [19]) directly produced from membrane. The proteins are kept in their native state with retained structure and function. The LPI™ FlowCell, allows for multiple rounds of chemical treatment and a wide variety of applications since the membrane vesicles are attached directly to the surface. The work-flow starts with the preparation of small membrane vesicles from *S*. Typhimurium. The membrane vesicles are washed and are then injected into the LPI™ FlowCell, allowing attachment to the surface. The immobilised membranes are then subjected to enzymatic digestion of proteins, in one or multiple steps to increase sequence coverage. By using proteases such as trypsin, the surface exposed parts of the membrane associated proteins are digested into smaller peptide fragments which can be eluted from the flow cell and analysed by liquid chromatography - tandem mass spectrometry (LC-MS/MS). A multi-step protocol can then be designed to increase the total sequence coverage of proteins identified, and so adding more confidence to the results generated using the LPI™ FlowCell. This approach allowed to identify a larger number of outer membrane proteins expressed by *S*. Typhimurium than previously reported [20] where many of which are associated with virulence.

## Results

### Preparation of outer membrane vesicles

A key step for the successful isolation of outer membrane proteins when using the LPI technology is the generation of outer membrane vesicles (OMVs). Here cells were converted into osmotically sensitive spheroplasts in triplicates by digesting the peptidoglycan layers of the cell wall with lysozyme. This was followed by osmotic shock treatment which induced the formation of vesicles at the outer membrane. Some were freely liberated as judged by electron microscopy. However, many were still attached to cells and were released by vigorous shaking. Intact, unbroken cells were removed from the vesicles by a low centrifugation step and the outer membrane vesicles were collected by ultracentrifugation.

**Figure 1:**
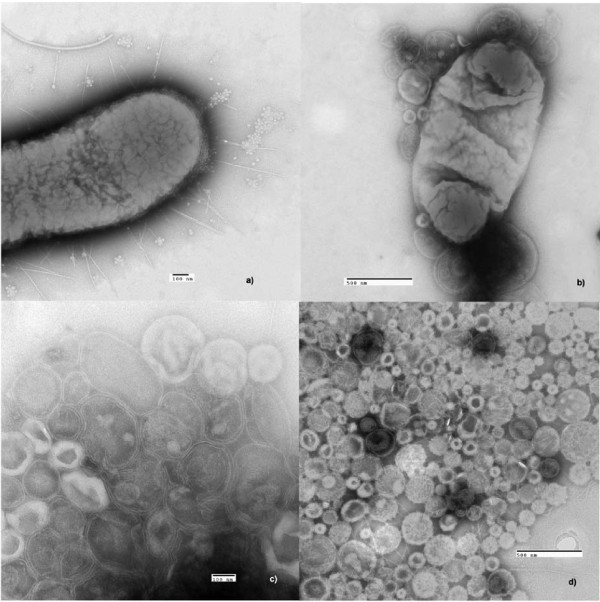
**Electron microscopy images illustrating the various stages of vesicle formation of Salmonella Typhimurium**. a) An intact washed Salmonella cell prior vesiculation treatment. b) a Salmonella cell during vesiculation of the outer membrane. c and d) Outer membrane vesicles.

The process of vesiculation and the purity of the vesicle suspension was monitored using electron microscopy (EM) (Figure 1). The various stages were monitored, that is from untreated washed cells to pure outer membrane vesicles to exclude as far as possible the presence of whole cells prior to loading on the LPI™ FlowCell. The images obtained by EM demonstrated the morphological changes the cell undergoes during the vesiculation process and the efficiency of the procedures used to generate OMVs.

### Protein identification

All samples were prepared in three biological replicates and multiple technical replicates. The proteins were considered successfully identified if they were present in at least two of the biological replicate samples with at least two peptides assigned per protein. In the case of protein MltC, OmpX and STM308, which was found in only one of the replicates the corresponding spectra were manually examined to confirm their correct identification

### Optimization of wash protocol

Initially, outer membrane vesicles (OMVs) were washed with HPLC grade water (Sigma-Aldrich) and loaded onto the LPI™ FlowCell in triplicates. The proteins of the OMVs were digested with trypsin and the resulting peptides were eluted from the LPI™ FlowCell and analysed using LC-MS/MS. In total, 301 proteins were identified of which 198 were identified with two or more peptide hits. Out of this 14 proteins (7%) were classified as outer membrane proteins (Table 1).

**Table 1:** Proteins identified in the first trypsin digest with and without a sodium carbonate wash step.

Protein type	Sample Group			
	
	HPLC grade water wash		Sodium Carbonate wash	
	
	Incl 1 peptide	>1 peptide	Incl 1 peptide	>1 peptide
All types	301	198	233	142

Non-membrane	253	168	134	81

Membrane-associated	48	30	99	61

OMP	26	14	54	42

% Non-membrane	84%	85%	58%	57%

% Membrane-assoc.	16%	15%	42%	43%

% OMP	9%	7%	23%	29%

The low proportion of outer membrane proteins was attributed to high level of contamination from cytosolic proteins. The washing protocol using HPLC grade water was considered not to be efficient in removing cytosolic proteins that were non-specifically attached to the membrane vesicles. To reduce the level of contamination, a further set of experiments were carried out where the vesicle preparations, in triplicates, were washed twice with ice cold sodium carbonate prior to being loaded onto the LPI™ FlowCell. In total, 233 proteins were identified of which 142 were identified with two or more peptide hits. The percentage of non-membrane associated proteins identified dropped from 85% to 57% when compared to the preparation without a sodium carbonate wash. The removal of cytosolic proteins was accompanied with an increase of the outer membrane proteins detected. After the washing step, 28 additional OMPs were detected giving a total of 42 OMPs identified with more than 1 peptide hit (Table 1). There was a four-fold increase in proportion of outer membrane proteins from 7% to 29% when compared to the run that was not subjected to the sodium carbonate wash step (Table 1).

### Optimization using multi-step protocols

Considering many of the outer membrane and membrane associated proteins were identified from a single peptide, the immobilised vesicles were subjected to a second round of trypsin digestion for 1 hr in order to generate additional peptides and increase the sequence coverage. In total, 122 proteins were identified of which 74 were identified with more than one peptide hit (Table 2).

**Table 2:** Number of proteins identified in the second digest with and without PPS Silent^® ^Surfactant

Protein type	Sample Group			
	
	No PPS		+ PPS	
	
	Incl 1 peptide	>1 peptide	Incl 1 peptide	>1 peptide
All types	122	74	162	89

Non-membrane	43	23	62	31

Membrane-associated	79	51	100	58

OMP	48	38	59	42

% Non-membrane	35%	31%	38%	35%

% Membrane-assoc.	65%	69%	62%	66%

% OMP	39%	51%	37%	47%

In an attempt to further maximise the sequence coverage, in duplicate, the immobilised vesicles were exposed to a second round of trypsin digestion for 1 hr with PPS Silent^®^, a reagent formulated for the extraction and solubilisation of hydrophobic peptides. PPS Silent^® ^is compatible with mass spectrometry and has been shown to improve the in-solution enzymatic digestions of hydrophobic proteins. As a result, a total of 162 proteins were identified of which 89 were identified with two or more peptide hits. In addition, the percentage of non membrane-associated proteins increased slightly from 31% to 35% when compared to the run without PPS Silent^®^. Further analysis, specifically for outer membrane proteins revealed that 42 (47%) of the proteins identified with two or more peptide hits were classified as outer membrane proteins. However, when compared to the digest without PPS Silent^® ^there was a small drop in the proportion of outer membrane proteins identified from 51% to 47% (Table 2), even though the number of outer membrane proteins increased from 38 to 42. The second digestion step resulted in a further 12 proteins being identified with two or more peptide hits (Additional file 1) where in some cases no peptides where found in the first digest.

Collating the results from both the first and second digests, a total of 54 outer membrane proteins were identified with two or more peptide hits with varying functions. Previous experiments performed by Coldham *et al *[20] identified 34 outer membrane proteins using a method based on a multi step fractionation strategy of the whole cell lysate into its various intracellular parts coupled with two dimensional HPLC-mass spectrometry (2D-LC-MS/MS). Here we identified 18 of the 34 outer membrane proteins which is summarised in Additional file 2. Furthermore, studies carried out by Molloy *et al *[13] identified 30 outer membrane proteins from *Escherichia coli (E. coli) *which is closely related to *S*. Typhimurium using sodium carbonate to enrich for outer membrane proteins and the detergent ASB-14 to solubilise them prior 2D GE. In this study we managed to identify 15 out of the 30 outer membrane proteins which is is summarised in Additional file 2. Outer membrane proteins identified included various transport proteins such as the vitamin B_12 _transporter BtuB precursor, long-chain fatty acid transport protein and the outer membrane usher protein, maltoporin as well as enzymes such as membrane-bound lytic murein transglycosylase C precursor, MltC. Furthermore, various proteins associated with virulence were also identified, such as major outer membrane lipoprotein 1 precursor, Lpp1 and outer membrane protease and receptor for phage OX2, OmpX which is essential for full virulence and survival within macrophages. A complete list of the outer membrane proteins identified together with their known biological functions are summarised in Additional file 1.

## Discussion

Membrane proteins are extremely difficult to isolate and characterise due to their association with the lipid bi-layer or the peptidoglycan and relatively lower abundance when in comparison with the whole cell complex. Established methods for the extraction and characterisation of membrane proteins that are commonly used include sodium carbonate precipitation, sucrose density gradients and the use of detergents to selectively solubilise and enrich the sample in favour of membrane proteins [8]. However these methods each have their own caveats. Detergent based methods use reagents that are often directly incompatible with downstream analytical techniques and so further clean up steps are required, resulting in a lengthy workflow [12,21] while sucrose density gradient and sodium carbonate precipitation face problems when resolubilising the membrane protein enriched fraction.

Here, we attempted to characterise the surface proteome of *S*. Typhimurium using Lipid-based Protein Immobilisation technology in the form of LPI™ FlowCells. The LPI™ FlowCell system provides a novel platform for the identification and characterisation of membrane proteins. No detergents are required and no sample clean up is needed prior to downstream analysis. The immobilised proteins can be digested with proteases in multiple steps to increase sequence coverage, and the peptides eluted can be characterised directly using LC-MS/MS.

Initial work highlighted the need to incorporate a wash step during the production of the intact membrane vesicles to minimise the carryover of contaminating cytosolic proteins that can potentially mask the lower abundant OMPs. The results generated showed that washing the membrane vesicles with a high pH sodium carbonate solution lowered the amount of non membrane proteins identified, and so enriching the vesicle preparation in favour of outer membrane proteins.

We have shown that a multi-step digest protocol can also be effectively used to increase total sequence coverage of proteins and to generate a list of outer membrane proteins identified with a greater confidence. However, even after incorporating a second digestion step, 17 outer membrane proteins were still only identified with one peptide hits, which is probably due to them being of low abundance. The addition of the acid cleavable mass spectrometry compatible detergent PPS Silent^® ^was incorporated into the work flow to try and improve the solubilisation and in-solution enzymatic protein digestions of hydrophobic proteins with trypsin. Results indicated that the addition of PPS Silent^® ^increased the total amount of different proteins identified with one peptide hits. However when counting just confident protein identifications (two or more peptide hits) this increase is less pronounced. Looking at confident protein identifications with PPS Silent^®^, the total number of outer membrane proteins increased from 38 to 42. However, PPS Silent^® ^appears to enhance detection of non-membrane proteins over outer membrane proteins as the proportion of non-membrane proteins increased marginally, while the proportion of outer membrane proteins decreased in the samples subjected to PPS Silent^®^. This suggests that outer membrane proteins are relatively resistant to solubilising in PPS Silent^®^, while non-membrane associated proteins solubilise more readily.

When comparing the data generated from this study with previously published work by Coldham & Woodward, more OMPs (total of 54) were identified here in comparison to 34 reported in their study. However, there were proteins that were not identified by using the LPI™ FlowCell. Coldham & Woodward[20] identified 34 outer membrane proteins using a method based on fractionating the whole cell lysate into its various intracellular parts coupled with two dimensional HPLC-mass spectrometry (2D-LC-MS/MS). Of the 34 outer membrane proteins identified, just over half (18) were found in our dataset. Overall there were 36 *S*. typhimurium OMPs identified in our dataset that were not reported previously [20] (Additional file 2). Some of these differences may be due to the use of different strains and variation in microbial culture conditions between both studies which will be reflected in their protein expression profiles. In addition, since the method used by Coldham & Woodward relied on multiple fractionation steps of the whole cell lysate, potential loss of outer membrane proteins, especially lower abundant ones could have occurred at each step in their workflow. Furthermore, it has been reported that results generated from mass spectrometry vary depending on the database search algorithm used to identify proteins [22]. The work carried out by Coldham &*Woodward *used the search algorithm SEQUEST, while in this study the search algorithm MASCOT was used. Therefore, the differences observed between the two methods could also be attributed to the database search algorithms and parameters used. Previous work carried out by Molloy *et al *[13] identified 30 outer membrane proteins from *Escherichia coli (E. coli) *which is closely related to *S*. Typhimurium using a method based on the enrichment of outer membrane proteins using sodium carbonate washes and incorporating the detergent ASB-14 to aid in solubilising them prior 2D GE. This study manages to identify 15 of the 30 outer membrane proteins. A further 15 outer membrane proteins reported by Molloy *et al *were not seen in this study while 39 outer membrane proteins were identified in this study that was not reported by Molloy *et al*. Some of these differences may be attributed again to the different strains and growth conditions used as well as the different instrument used to identify the proteins and bioinformatics tools used to confirm the presence of outer membrane proteins. These results show there is no real consensus of proteins identified between the LPI™ FlowCell method and more established methods such as 2D GE and 2D-LC-MS/MS (Additional file 2). Instead these methods complement each other and therefore when designing experiments to identify outer membrane proteins it is important to try a range of approaches to maximise the coverage of OMPs detected.

Finally, when collating the results from both digests performed in this study, different classes of membrane proteins with varying functions were also identified. A total of 69 proteins were identified as being outer membrane proteins of which 54 were identified with two or more peptide hits (Additional file 1). Using the database UniProtKB http://www.uniprot.org some of the functions of the outer membrane proteins were deduced. These included the transporters BtuB which is responsible for the uptake of vitamin B_12_, LamB which is involved in the uptake of maltose and maltodextrins and LolB which is involved in the incorporation of lipoproteins in the outer membrane. Other biologically significant proteins identified included the enzymes MltC which may play a role in cell elongation and division and NlpD which is involved in catabolic processes in cell wall formation as well as proteins involved in virulence such as Lpp1, Lpp2 and OmpX. To further verify the functions of the outer membrane proteins identified in the present study, manual mining of the data, which involved searching through literature containing information on the proteins of interest, was also undertaken. This approach shed further light on outer membrane proteins identified that were not apparent using UniProtKB, a shortcoming of using a single approach to verify the functions of proteins [23]. These included membrane-bound lytic murein transglycosylase (MltB and MltC) which is important for cell growth [24], conjugal transfer surface exclusion protein (TraT) which is responsible for resistance to bacterial killing by serum [25] and RcsF protein which is part of the Rcs phosphorelay signalling pathway responding to peptidoglycan damage by regulating colanic acid capsular exopolysaccharide synthesis, and has also been seen to enhance bacterial survival in the presence of antibiotics [26].

## Conclusions

The present study aimed to elucidate the expression of outer membrane proteins in *Salmonella *Typhimurium using LPI™ FlowCells. The membrane preparations largely excluded most of the cytosolic proteins that co-purifies with it when using currently available fractionation procedures and therefore achieved a wider coverage of the membrane subproteome than had been reported. Furthermore, since there was no real cross over in the outer membrane proteins identified between this method than previously reported, highlights the importance of incorporating a range of methods into the experimental design that complement each other to maximise the range of outer membrane proteins identified. In addition, it has been emphasised frequently, that while downstream analysis of proteins have improved markedly over the last decade with ever increasing mass spectral analysis and software developments, initial sample preparation methods from various microorganisms and fractionation procedures, particularly for low abundant proteins have lagged behind. Several approaches are being used, one of the most recent being the use of combinational peptide libraries. The technique was used successfully to study cell extracts of *E. coli *and resulted in a significant increase in the number of proteins that are normally detected and included very low copy number metabolic enzymes [27]. A drawback of this approach is the large volume of starting material required. It is our view based on current sub-cellular fractionation procedures, that LPI™ technology currently provides the widest coverage of outer membrane proteins as demonstrated here for *Salmonella *Typhimurium. Current studies are aimed at culturing this microorganism in growth conditions more akin to those *in vivo *to gain further insight into the expression of the membrane proteins and the role of specific proteins in disease.

## Methods

### Bacterial strain and culture conditions

Salmonella *enterica *serovar Typhimurium LT2 (ATCC 700720) was grown aerobically on nutrient broth in triplicate at 37°C with constant shaking at 200 rpm. Bacterial cells from a 500 ml culture were collected in stationary phase (OD_600 _= 1.2-1.5) via centrifugation at 13 000 ***g ***at 4°C for 40 min. The collected cells were washed 3 times with phosphate buffered saline (PBS; pH 7) and stored at -80°C for further use.

### Preparation of outer membrane vesicles

The following method was adapted from Kaback (1971) [28]. The harvested cells were washed three times with Tris buffer containing 20% sucrose (w/v) (Fluka), 30 mM Tris-HCl (GE Healthcare) and 10 mM EDTA (Fluka) at pH 8.0 and collected by centrifugation at 21 000 ***g ***for 40 min at 4°C. The washed cells were resuspended in 10 ml Tris/sucrose buffer containing 5 mg ml^-1 ^lysozyme (Sigma Aldrich), and incubated at room temperature for 45 min with gentle shaking. The spheroplasts produced by this procedure were harvested by centrifugation at 21 000 ***g ***for 30 min at 4°C. The pellet containing the spheroplasts was resuspended in 10 ml of 10 mM phosphate buffer (pH 7) containing 2 mM MgSO_4 _(Sigma Aldrich), 10 mg ml^-1 ^ribonuclease A (Sigma Aldrich) and 10 mg ml^-1 ^deoxyribonuclease I (Sigma Aldrich) and incubated at 37°C for 45 min with vigorous shaking. During this step the osmotically induced vesicles on the cell surface detach from the cells (Figure. 1). The unbroken cells were removed by centrifugation at 1000 ***g***, 30 min, 4°C and the supernatant containing the membrane vesicles was kept. The membrane vesicles were collected by ultracentrifugation at 115 000 ***g***, 1 h, 4°C followed by two wash steps by resuspending them in 200 mM Na_2_CO_3 _(Sigma-Aldrich) and placing them in an ultrasonic bath on ice for 30 min. The high pH and high salt concentration facilitates the removal of cytosolic proteins. The washed membrane vesicles were resuspended using a pipette in 600 μl Tris-buffer containing high salt concentration of sodium chloride (10 mM Tris-HCl, 300 mM NaCl, pH 8) and stored at -80°C.

### Electron microscopy

Electron microscopy was carried out to confirm that membrane vesicles were present and that no whole cells have been carried over prior to running the sample on the LPI™ FlowCells. Vesicle preparations (100 μl) were inactivated by adding Carson's buffered formalin (Bios Europe Ltd) to give a final concentration of 1% (v/v) formaldehyde in the vesicle suspension. The inactivated suspension was made up to 1 ml with distilled water and centrifuged at 48 000 *g *for 45 minutes. The supernatant was discarded and the pellet re-suspended in 25 μl distilled water. Five μl of re-suspended pellet was mixed with 5 μl 1% (v/v) potassium phosphotungstic acid (PTA) containing 0.05% (v/v) bovine serum albumin. A 400 mesh formvar-carbon coated copper EM grid was floated on the drop for several minutes and was then blotted by touching a piece of filter paper to the edge of the grid. Grids were examined in a Philips 420 transmission electron microscope.

### Operation of the LPI™ FlowCells - single trypsin digestion

A solution containing outer membrane vesicles from *S*. Typhimurium (500 μl) was injected into the LPI™ FlowCell followed by incubation at room temperature for 1 h. This allowed the vesicles to attach to the membrane-attracting surfaces. The LPI™ FlowCell was rinsed with 2 ml of 10 mM Tris-HCl containing 300 mM NaCl at pH 8.0, followed by 2 ml of 20 mM ammonium-bicarbonate buffer (NH_4_HCO_3_), pH 8.0 and incubated at 37°C for 10 min. Seven hundred μl of 20 mM NH_4_HCO_3 _containing 5 μg ml^-1 ^trypsin (sequencing grade, Promega) was injected into the LPI™ FlowCell and incubated at 37°C for 2 h. The resulting peptides were collected from the LPI™ FlowCell by injecting 700 μl of 20 mM NH_4_HCO_3_, pH 8.0 at the inlet port and concomitantly capturing the eluted liquid at the outlet port. Fourteen μl of formic acid was added to the captured peptides to inactivate the trypsin and the sample was stored at -80°C for further use.

### Operation of the LPI™ FlowCells - multi-step digestion

Trypsin was used for the first digestion step and the sample was digested for 30 minutes as described above for single trypsin injection. After elution of the peptides a second step digestion was performed on the captured stationary membrane vesicles in the LPI™ FlowCell. For the second digestion step, 700 μl of 20 mM NH_4_HCO_3 _containing 5 μg ml^-1 ^of trypsin, pH 8.0 was injected into the LPI™ FlowCell and then incubated at 37°C for 1 h. The tryptic peptides were collected as described above for single trypsin injection.

### Operation of the LPI™ FlowCells - multi-step digestion with PPS Silent^® ^Surfactant

PPS Silent^® ^Surfactant (Protein Discovery) is a mass spectrometry compatible reagent designed for the extraction and solubilisation and improvement of in-solution enzymatic protein digestions of hydrophobic proteins.

For the first digestion step with trypsin, the same procedure was followed as for the multi-step digestion method without PPS Silent^® ^Surfactant as described above.

For the second digestion step, trypsin was resuspended in 20 mM NH_4_HCO_3 _pH 8.0 to a final concentration of 5 μg ml^-1^. The resuspended trypsin was then used to resuspend PPS Silent^® ^Surfactant to a final concentration of 0.1% (w/v). 700 μl of the trypsin containing PPS Silent^® ^Surfactant was then injected into the LPI™ FlowCell and then incubated at 37°C for 1 h. The tryptic peptides were collected by injecting 700 μl 20 mM NH_4_HCO_3_, pH 8 at the inlet port and collecting the eluant at the outlet port. Formic acid was added to the eluted peptides to a final concentration of 250 mM and incubated for 1 h at room temperature to inactivate the trypsin and cleave the PPS Silent^® ^Surfactant from the sample. The sample was stored at -80°C for further analysis (see Additional File 3).

### Peptide analysis using liquid chromatography tandem mass spectrometry (LC-MS/MS)

The peptide fraction collected from LPI™ FlowCell was subsequently analyzed separately by LC- MS/MS at the Proteomics Core Facility at the University of Gothenburg. Prior to analysis, the sample was centrifuged in vacuum to dryness and reconstituted in 20 μl 0.1% (v/v) formic acid in water. The sample was centrifuged at 13 000 ***g ***for 15 minutes and 17 μl was transferred to the autosampler of the LC-MS/MS system. For the liquid chromatography, an Agilent 1100 binary pump was used and the tryptic peptides were separated on a 200 × 0.05 mm i.d. fused silica column packed in-house with 3 μm ReproSil-Pur C18-AQ particles (Dr. Maisch, GmbH, Ammerbuch, Germany). Two μl of the sample was injected and the peptides were first trapped on a precolumn (45 × 0.1 mm i.d.) packed with 3 μm C18-bonded particles. A 40 minute gradient of 10-50% (v/v) acetonitrile in 0.2% (v/v) formic acid was used for separation of the peptides. The flow through the column was reduced by a split to approximately 100 nl min^-1^. Mass analyses were performed in a 7-Tesla LTQ-FT mass spectrometer (Hybrid Linear Trap Quadrupole - Fourier Transform; Thermo Electron) equipped with a nanospray source modified in-house. The instrument was operated in the data-dependent mode to automatically switch between MS and MS/MS acquisition. MS spectra were acquired in the FT-ICR while MS/MS spectra were acquired in the LTQ-trap. For each scan of FT-ICR, the six most intense, double- or triple protonated ions were sequentially fragmented in the linear trap by collision induced dissociation (CID). Already fragmented target ions were excluded for MS/MS analysis for 6 seconds. All tandem mass spectra were searched by MASCOT (Matrix Science) against the bacterial database Uniprot http://www.uniprot.org. Search parameters specified an initial peptide mass tolerance of +/- 5 ppm, an MS/MS tolerance of +/- 0.5 Da and full trypsin specificity allowing for up to 1 missed cleavages. Oxidation of methionine were set as variable modification.

### Detection of Outer Membrane Proteins (OMP's)

An in-house database was curated containing the results from seven sub-cellular predictor programs (LIPO [29], LipoP v1.0 [30], Proteome Analyst v2.5 [31], CELLO v2.5 [32], PSORTb v2.0 [33,34], TMHMM v2.0 [35,36] and BOMP [37]) as well as using the data obtained from the Gene Ontology Annotation (GOA) database [38,39], Gene Ontology database GOOSE http://www.berkeleybop.org/goose and UniProtKB http://www.uniprot.org/help/uniprotkb. Experimental data acquired by Coldham *et al*. [20] where 34 outer membrane proteins were identified in *Salmonella *Typhimurium was also added to the database. Proteins were identified as outer membrane proteins if 1) the protein name suggests outer membrane, or, 2) if any of the GOA, GOOSE, UniProtKB, BOMP, PSORTb or the experimental data obtained by Coldham *et al*. indicate outer membrane, or, 3) Both Proteome Analyst and CELLO predicts the presence of outer membrane proteins, or, 4) if both LIPO and LipoP predicts the presence of lipoproteins.

## Competing interests

RK was previously employed by Nanoxis AB and therefore received salary during the last 5 years. RK has shares in Nanoxis AB as he is a co-founder. RK is a co-author of a patentdescribing the LPI-technologythat is owned by Nanoxis AB. RK has no other financial interests. Rk has no non-financial competing interests. The authors DC, VE, HNS, CA and HA declare that they have no competing interests.

## Authors' contributions

DC carried out the growth, preparation and digests of vesicles of *S*. Typhimurium. HA performed the electron microscopy analysis of the vesicle preparations. RK performed the mass spectrometry identification and data mining of the proteins. VE and HNS participated in the design of the study. HNS conceived and coordinated the study. All authors read and approved the final manuscript.

## Supplementary Material

Additional file 1 **Outer membrane proteins identified and number of peptides generated using a single or multi-step digest protocol**. Table listing outer membrane proteins identified from single and multi-step digest protocols after using the LPI™ FlowCell Click here for file

Additional file 2 **Comparison of the outer membrane proteins identified in this study with that reported by Coldham & Woodward and Molloy *et al***. Table comparing the results from this study with that reported by Coldham &*Woodward *and Molloy *et al*. Click here for file

Additional file 3 **Flow diagram showing the basic steps in operating a LPI™ FlowCell**. A flow diagram showing the main steps in using the LPI™ FlowC Click here for file
