# Supplementary material for: Elucidation of the outer membrane proteome of Salmonella enterica serovar Typhimurium utilising a lipid-based protein immobilization technique
Source: BMC Microbiol. 2010 Feb 11;10:44. doi: 10.1186/1471-2180-10-44 (PMC2829538; doi:10.1186/1471-2180-10-44)
Supplement: Additional file 1 — Outer membrane proteins identified and number of peptides generated using a single or multi-step digest protocol. Table listing outer membrane proteins identified from single and multi-step digest protocols after using the LPI™ FlowCell [file 1471-2180-10-44-S1.DOC]

Additional file 1: Outer membrane proteins identified and number of peptides generated using a single or multi-step digest protocol

Bolded identifications highlight the ability of multi-step approach to generate further unique peptides of a particular protein and so increasing its sequence coverage and ultimately increasing the list of two or more peptide hit identifications.

^*^ Outer membrane protein also identified by previous work by Coldham *et al* [20]

| Protein | Name | Number of peptides from single trypsin digestion step | Number of peptides from multi-step digestion | Combined number of unique peptides from first and second digest | Biological process (Uniprot annotation) | Biological function (Uniprot annotation) |
| --- | --- | --- | --- | --- | --- | --- |
| **MULTIPLE PEPTIDE IDENTIFICATION** | | | | | | |
| BtuB^*^ | Vitamin B12 transporter btuB precursor | 3 | 14 | 14 | Ion transport | Translocation of vitamin B12 across the outer membrane |
| FadL^*^ | Long-chain fatty acid transport protein | 5 | 4 | 6 | Lipid transport | Translocation of long-chain fatty acids across the outer membrane |
| FimD | Outer membrane usher protein | 9 | 19 | 19 | Transport | Involved in the export and assembly of fimA fimbrial subunits across the outer membrane |
| Imp^*^ | LPS-assembly protein precursor | 17 | 11 | 21 |  |  |
| **LamB^*^** | **Maltoporin** | **0** | **2** | **2** | Ion transport Sugar transport | Involved in the transport of maltose and maltodextrins |
| LolB | Outer-membrane lipoprotein lolB precursor | 3 | 4 | 6 | Protein transport | Involved in the incorporation of lipoproteins in the outer membrane |
| Lpp1 | Major outer membrane lipoprotein 1 precursor | 7 | 7 | 7 | Virulence | Interacts with the peptidoglycan both covalently and noncovalently. Plays an important role in virulence |
| Lpp2 | Major outer membrane lipoprotein 2 precursor | 2 | 2 | 2 | Virulence | Interacts with the peptidoglycan both covalently and noncovalently. Plays an important role in virulence. |
| MetQ | D-methionine-binding lipoprotein metQ precursor | 2 | 4 | 4 | Amino acid transport | Component of a D-methionine permease, a binding protein-dependent, ATP-driven transport system |
| **MltB** | **Membrane-bound lytic murein transglycosylase B** | **0** | **3** | **3** | Enzyme | glycosidase hydrolase |
| MltC | Membrane-bound lytic murein transglycosylase C precursor | 2 | 1 | 2 | Enzyme | Murein-degrading enzyme. May play a role in recycling of muropeptides during cell elongation and/or cell division |
| NlpB^*^ | Lipoprotein-34 | 7 | 15 | 15 |  |  |
| NlpD | Lipoprotein | 5 | 6 | 6 | Enzyme | involved in catabolic processes |
| **NlpI** | **Lipoprotein, cell division** | **1** | **3** | **3** |  |  |
| OmpA^*^ | Outer membrane protein A | 19 | 21 | 24 | Conjugation Ion transport Phage recognition | Required for the action of colicins K and L and for the stabilization of mating aggregates in conjugation. Serves as a receptor for a number of T-even like phages. Also acts as a porin with low permeability that allows slow penetration of small solutes |
| OmpD | Outer membrane porin protein ompD precursor | 4 | 6 | 6 | Ion Transport |  |
| **OmpW** | **Outer membrane protein W precursor** | **1** | **5** | **5** |  |  |
| **OmpX** | **Outer membrane protease, receptor for phage OX2** | **1** | **3** | **3** | Virulence | Essential for full virulence and survival within macrophages |
| OsmE | Osmotically inducible lipoprotein E | 2 | 3 | 3 |  |  |
| Pal^*^ | Peptidoglycan-associated lipoprotein | 4 | 8 | 8 |  |  |
| RcsF | RcsF protein | 2 | 3 | 3 |  |  |
| RlpA | Minor lipoprotein | 9 | 9 | 11 |  |  |
| RlpB | LPS-assembly lipoprotein rlpB precursor | 5 | 6 | 9 |  | involved in the assembly of LPS in the outer leaflet of the outer membrane |
| **Skp** | **Chaperone protein skp precursor** | **1** | **2** | **2** |  |  |
| Slp^*^ | Putative lipoprotein | 4 | 6 | 6 |  |  |
| SlyB^*^ | Outer membrane lipoprotein SlyB | 3 | 5 | 5 |  |  |
| Ssb | Single-stranded DNA-binding protein | 2 | 0 | 2 |  |  |
| STM0920 | Fels-1 prophage attachment and invasion protein | 2 | 2 | 3 |  |  |
| STM1607^*^ | Putative outer membrane lipoprotein | 2 | 4 | 4 |  |  |
| **STM2447** | **Putative outer membrane lipoprotein** | **1** | **2** | **2** |  |  |
| **STM3038** | **Putative metalloendopeptidase** | **1** | **1** | **2** |  |  |
| TolC^*^ | Outer membrane channel | 8 | 8 | 10 |  |  |
| TraT | Conjugative transfer: surface exclusion | 2 | 6 | 6 | Conjugation | Responsible for preventing unproductive conjugation between bacteria carrying like plasmids |
| Tsx^*^ | Nucleoside channel; receptor of phage T6 and colicin K | 6 | 11 | 11 | Ion transport Phage recognition | part of the receptor for colicin K and phage T6, and functions as substrate-specific channel for nucleosides and deoxynucleosides |
| VacJ^*^ | Lipoprotein | 5 | 6 | 6 |  |  |
| YaeT^*^ | Outer membrane protein assembly factor yaeT precursor | 17 | 16 | 23 |  | Involved in the assembly of outer membrane proteins |
| YajG | Putative lipoprotein | 3 | 4 | 4 |  |  |
| YajI | Putative lipoprotein | 2 | 2 | 2 |  |  |
| **YbaY** | **Conserved hypothetical lipoprotein** | **1** | **3** | **4** |  |  |
| YbhC^*^ | Possible pectinesterase | 5 | 10 | 10 |  |  |
| YbjP | Putative lipoprotein | 2 | 3 | 3 |  |  |
| **YbjR** | **Putative aminidase** | **0** | **3** | **3** |  |  |
| **YceB** | **Putative outer membrane lipoprotein** | **1** | **2** | **2** |  |  |
| YcfM | Putative lipoprotein | 2 | 4 | 4 |  |  |
| YedD^*^ | Putative lipoprotein | 5 | 6 | 7 |  |  |
| YfgL | Putative lipoprotein | 8 | 15 | 15 |  |  |
| YfiO | Putative lipoprotein | 8 | 14 | 14 |  |  |
| YggG | Putative uncharacterized protein yggG | 2 | 3 | 4 |  |  |
| YiaD^*^ | Putative outer membrane lipoprotein | 3 | 7 | 7 |  |  |
| **YjfO** | **Putative exported protein** | **1** | **2** | **2** |  |  |
| YmbA^*^ | Putative lipoprotein | 2 | 3 | 3 |  |  |
| YraM | Paral putative transglycosylase | 7 | 11 | 13 |  |  |
| YraP | Paral putative periplasmic protein | 7 | 9 | 9 |  |  |
| YtfM | Putative exported protein | 3 | 2 | 4 |  |  |
| **SINGLE PEPTIDE IDENTIFICATION** | | | | | | |
| EcnB | Entericidin B | 1 | 1 | 1 |  |  |
| EnvE | Putative lipoprotein | 0 | 1 | 1 |  |  |
| FimH | Fimbrial subunit | 0 | 1 | 1 | Fimbrium | Involved in regulation of length and mediation of adhesion of type 1 |
| MipA | Putative outer membrane protein | 1 | 1 | 1 |  |  |
| OmpC^*^ | Outer membrane protein C | 0 | 1 | 1 | Ion transport Phage recognition | Forms passive diffusion pores which allow small molecular weight hydrophilic materials across the outer membrane |
| OsmB | Osmotically inducible lipoprotein B | 0 | 1 | 1 |  | Provides resistance to osmotic stress. May be important for stationary-phase survival |
| PagP | Antimicrobial peptide resistance and lipid A acylation protein | 0 | 1 | 1 |  |  |
| SmpA | Small membrane protein A | 1 | 1 | 1 |  |  |
| SopB^*^ | Inositol phosphate phosphatase sopB | 0 | 1 | 1 |  |  |
| STM1940 | Putative cell wall-associated hydrolase | 0 | 1 | 1 |  |  |
| YaoF | Putative hemolysin | 0 | 1 | 1 |  |  |
| YgdI | Putative lipoprotein | 0 | 1 | 1 |  |  |
| YgdR | Possible lipoprotein | 0 | 1 | 1 |  |  |
| YifL^*^ | Uncharacterized lipoprotein yifL precursor | 1 | 1 | 1 |  |  |
| YjeI^*^ | Putative outer membrane lipoprotein | 0 | 1 | 1 |  |  |
