# Supplementary material for: Elucidation of the outer membrane proteome of Salmonella enterica serovar Typhimurium utilising a lipid-based protein immobilization technique
Source: BMC Microbiol. 2010 Feb 11;10:44. doi: 10.1186/1471-2180-10-44 (PMC2829538; doi:10.1186/1471-2180-10-44)
Supplement: Additional file 2 — Comparison of the outer membrane proteins identified in this study with that reported by Coldham & Woodward and Molloy et al. Table comparing the results from this study with that reported by Coldham &Woodward and Molloy et al. [file 1471-2180-10-44-S2.DOC]

Additional file 2: Comparison of the outer membrane proteins identified in this study with that reported by Coldham & Woodward and Molloy *et al.*

| **Protein** | **Name** | **Found in Coldham *& Woodward* [20]** | **Found in Molloy *et al* [13]** | **Found in this study** |
| --- | --- | --- | --- | --- |
| FliC | Flagellin | ✓ | ✓ |  |
| OmpA | Putative hydrogenase, membrane component | ✓ | ✓ | ✓ |
| NmpC | New outer membrane protein; predicted bacterial porin | ✓ |  |  |
| SlyB | Putative outer membrane lipoprotein | ✓ | ✓ | ✓ |
| OmpC | Outer membrane protein 1b (ib;c), porin | ✓ | ✓ | ✓ (1 peptide) |
| NlpB | Lipoprotein-34 | ✓ | ✓ | ✓ |
| Pal | Tol protein required for outer membrane integrity, uptake of group Ac | ✓ | ✓ | ✓ |
| YaeT | Putative outer membrane antigen | ✓ |  | ✓ |
| YiaF | Putative outer membrane lipoprotein | ✓ |  |  |
| TolC | Outer membrane channel; specific tolerance to colicin E1 | ✓ | ✓ | ✓ |
| HlpA | Histone-like protein, located in outer membrane | ✓ |  |  |
| YbhC | Putative pectinesterase | ✓ | ✓ | ✓ |
| Imp | Organic solvent tolerance protein | ✓ |  | ✓ |
| Tsx | Nucleoside channel; receptor of phage T6 and colicin K | ✓ | ✓ | ✓ |
| LamB | Phage *ì* receptor protein; maltose high-affinity receptor | ✓ | ✓ | ✓ |
| OmpS | Putative porin | ✓ |  |  |
| YiaD | Putative outer membrane lipoprotein | ✓ |  | ✓ |
| YedD | Putative outer membrane lipoprotein | ✓ |  | ✓ |
| YjeI | Putative outer membrane lipoprotein | ✓ |  | ✓ (1 peptide) |
| OmpF | Outer membrane protein 1a (ia;b;f), porin | ✓ | ✓ |  |
| BtuB | Outer membrane receptor for transport of vitamin B12, E colicins | ✓ | ✓ | ✓ |
| FhuA | Outer membrane protein receptor/transporter for ferrichrome, colicin | ✓ | ✓ |  |
| SopB | Salmonella outer protein: homologous to ipgD of Shigella | ✓ |  | ✓ (1 peptide) |
| MltA | Membrane-bound lytic murein transglycosylase A | ✓ |  |  |
| FadL | Transport of long-chain fatty acids; sensitivity to phage T2 | ✓ | ✓ | ✓ |
| InvG | Invasion protein; outer membrane | ✓ |  |  |
| VacJ | Lipoprotein precursor | ✓ |  | ✓ |
| YaeC | Putative outer membrane lipoprotein | ✓ |  |  |
| YifL | Putative outer membrane lipoprotein | ✓ |  | ✓ (1 peptide) |
| STM1607 | Putative outer membrane lipoprotein | ✓ |  | ✓ |
| YmbA | Putative outer membrane protein | ✓ |  | ✓ |
| FepA | Outer membrane porin, receptor for ferric enterobactin | ✓ | ✓ |  |
| Slp | Putative outer membrane protein | ✓ | ✓ | ✓ |
| YhfL | Putative outer membrane lipoprotein | ✓ |  |  |
| FimD | Outer membrane usher protein |  |  | ✓ |
| LolB | Outer-membrane lipoprotein lolB precursor |  |  | ✓ |
| Lpp1 | Major outer membrane lipoprotein 1 precursor |  |  | ✓ |
| Lpp2 | Major outer membrane lipoprotein 2 precursor |  |  | ✓ |
| MetQ | D-methionine-binding lipoprotein metQ precursor |  |  | ✓ |
| MltB | Membrane-bound lytic murein transglycosylase B |  |  | ✓ |
| MltC | Membrane-bound lytic murein transglycosylase C precursor |  |  | ✓ |
| NlpD | Lipoprotein |  |  | ✓ |
| NlpI | Lipoprotein, cell division |  |  | ✓ |
| OmpD | Outer membrane porin protein ompD precursor |  |  | ✓ |
| OmpW | Outer membrane protein W precursor |  | ✓ | ✓ |
| OmpX | Outer membrane protease, receptor for phage OX2 |  | ✓ | ✓ |
| OsmE | Osmotically inducible lipoprotein E |  | ✓ | ✓ |
| RcsF | RcsF protein |  |  | ✓ |
| RlpA | Minor lipoprotein |  |  | ✓ |
| RlpB | LPS-assembly lipoprotein rlpB precursor |  |  | ✓ |
| Skp | Chaperone protein skp precursor |  |  | ✓ |
| Ssb | Single-stranded DNA-binding protein |  |  | ✓ |
| STM0920 | Fels-1 prophage attachment and invasion protein |  |  | ✓ |
| STM2447 | Putative outer membrane lipoprotein |  |  | ✓ |
| STM3038 | Putative metalloendopeptidase |  |  | ✓ |
| TraT | Conjugative transfer: surface exclusion |  |  | ✓ |
| YajG | Putative lipoprotein |  |  | ✓ |
| YajI | Putative lipoprotein |  |  | ✓ |
| YbaY | Conserved hypothetical lipoprotein |  |  | ✓ |
| YbjP | Putative lipoprotein |  | ✓ | ✓ |
| YbjR | Putative aminidase |  |  | ✓ |
| YceB | Putative outer membrane lipoprotein |  |  | ✓ |
| YcfM | Putative lipoprotein |  |  | ✓ |
| YfgL | Putative lipoprotein |  |  | ✓ |
| YfiO | Putative lipoprotein |  |  | ✓ |
| YggG | Putative uncharacterized protein yggG |  |  | ✓ |
| YjfO | Putative exported protein |  |  | ✓ |
| YraM | Paral putative transglycosylase |  |  | ✓ |
| YraP | Paral putative periplasmic protein |  |  | ✓ |
| YtfM | Putative exported protein |  |  | ✓ |
| EcnB | Entericidin B |  |  | ✓ (1 peptide) |
| EnvE | Putative lipoprotein |  |  | ✓ (1 peptide) |
| FimH | Fimbrial subunit |  |  | ✓ (1 peptide) |
| MipA | Putative outer membrane protein |  |  | ✓ (1 peptide) |
| OsmB | Osmotically inducible lipoprotein B |  |  | ✓ (1 peptide) |
| PagP | Antimicrobial peptide resistance and lipid A acylation protein |  |  | ✓ (1 peptide) |
| SmpA | Small membrane protein A |  |  | ✓ (1 peptide) |
| STM1940 | Putative cell wall-associated hydrolase |  |  | ✓ (1 peptide) |
| YaoF | Putative hemolysin |  |  | ✓ (1 peptide) |
| YgdI | Putative lipoprotein |  |  | ✓ (1 peptide) |
| YgdR | Possible lipoprotein |  |  | ✓ (1 peptide) |
| FhuE | FhuE receptor |  | ✓ |  |
| CirA | Colicin I receptor |  | ✓ |  |
| YbiL | Catecholate siderophore receptor fiu |  | ✓ |  |
| AG43 (beta) | Antigen 43 beta chain |  | ✓ |  |
| AG43 (alpha) | Antigen 43 alpha chain |  | ✓ |  |
| OmpT | Protease 7 |  | ✓ |  |
| OmpP | Outer membrane protease ompP |  | ✓ |  |
| Pa1 | Phospholipase A1 |  | ✓ |  |
| YeaF | MltA-interacting protein |  | ✓ |  |
| UP05 | Unknown protein |  | ✓ |  |
