# Supplementary material for: Elucidation of the outer membrane proteome of Salmonella enterica serovar Typhimurium utilising a lipid-based protein immobilization technique
Source: BMC Microbiol. 2010 Feb 11;10:44. doi: 10.1186/1471-2180-10-44 (PMC2829538; doi:10.1186/1471-2180-10-44)
Supplement: Additional file 3 — Flow diagram showing the basic steps in operating a LPI™ FlowCell. A flow diagram showing the main steps in using the LPI™ FlowC [file 1471-2180-10-44-S3.DOC]

Additional file 3: Flow diagram showing the basic steps in operating a LPI^TM^ FlowCell

Membrane vesicles

Inject sample into LPI™ FlowCell & incubate @ RT for 1 hour

Rinse with 2 ml:

10 mM Tris-HCl pH 8.0

300 mM NaCl

incubate @ 37^o^C 10 min with 2 ml:

20 mM NH_4_HCO_3_ pH 8.0

incubate @ 37^o^C 1 hr min with 2 ml:

20 mM NH_4_HCO_3_ pH 8.0

5 μg/ml trypsin

Inject 700 µl NH_4_HCO_3_

Collect peptides

Add 14 μl formic acid & analyse by LC-MS/MS

incubate @ 37^o^C 1 hr min with 2 ml:

20 mM NH_4_HCO_3_ pH 8.0

5 μg/ml trypsin

Inject 700 µl NH_4_HCO_3_

Collect peptides

Add 14 μl formic acid & analyse by LC-MS/MS
